# Applying results from clinical trials: tranexamic acid in trauma patients

**DOI:** 10.1186/s40560-014-0056-1

**Published:** 2014-10-05

**Authors:** Ian Roberts, David Prieto-Merino

**Affiliations:** Clinical Trials Unit, London School of Hygiene & Tropical Medicine, Keppel Street, London, WC1E 7HT UK

**Keywords:** Evidence-based medicine, Generalizability, Clinical trials, Tranexamic acid, Trauma

## Abstract

This paper considers how results from clinical trials should be applied in the care of patients, using the results of the Clinical Randomisation of an Antifibrinolytic in Significant Haemorrhage (CRASH-2) trial of tranexamic acid in bleeding trauma patients as a case study. We explain why an understanding of the mechanisms of action of the trial treatment, and insight into the factors that might be relevant to this mechanism, is critical in order to properly apply (generalise) trial results and why it is not necessary that the trial population is representative of the population in which the medicine will be used. We explain why cause (mechanism)-specific mortality is more generalizable than all-cause mortality and why the risk ratio is the generalizable measure of the effect of the treatment. Overall, we argue that a biological insight into how the treatment works is more relevant when applying research results to patient care than the application of statistical reasoning.

## Introduction

In September 1962, writing in the Keio Journal of Medicine, Japanese researchers Shosuke and Utako Okamoto reported the invention of a new chemical entity that inhibited the enzymatic breakdown of fibrin by plasmin [1]. Initially referred to as AMCHA, this drug is now known as tranexamic acid. Tranexamic acid is a synthetic analogue of the amino acid lysine. It can be administered orally or by a short intravenous infusion after which peak plasma concentrations of tranexamic acid are obtained rapidly. It is excreted as unchanged drug in the urine with an elimination half-life of about 3 h.

The Clinical Randomisation of an Antifibrinolytic in Significant Haemorrhage (CRASH-2) trial was a randomised controlled trial of the effect of tranexamic acid on death and vascular occlusive events in bleeding trauma patients. The results were published in 2010 [2]. A total of 20,211 adult trauma patients with significant bleeding, who were within 8 h of their injury, were randomly allocated to receive tranexamic acid (TXA, 1 g over 10 min followed by an infusion of 1 g over 8 h) or matching placebo. The primary outcome was death in the hospital within 4 weeks. TXA significantly reduced death due to bleeding (RR = 0.85, 95% CI 0.76–0.96) and all-cause mortality (RR = 0.91, 95% CI 0.85–0.97), with no increase in vascular occlusive events. The reduction in death due to bleeding was greatest when TXA was given within 3 h of injury, (RR = 0.72, 95% CI 0.63–0.83) [3]. When TXA was given after 3 h, there was no mortality reduction. On the basis of these results, TXA was added to the WHO List of Essential Medicines and included in trauma protocols around the world.

If clinical trial results could not be applied (generalised) to people who did not take part in the trial, there would be little reason to do them. If a trial shows convincingly that a treatment reduces the risk of an adverse health outcome in one group of patients, we have to consider what effect it might have in another group of patients. Since the publication of the CRASH-2 results, there has been considerable discussion about how TXA should be used in practice. Some authors, pointing out that most of the patients in the CRASH-2 trial were recruited from hospitals in Africa, Asia and Latin America, question whether the results are applicable to ‘modern’ trauma care systems and call for further trials before implementing the results [4]. Others suggest limiting TXA use to specific patient subgroups, such as those with low blood pressure or laboratory evidence of ‘hyperfibrinolysis’ [5,6]. This paper considers some pathophysiological and epidemiological issues relevant to the application of the CRASH-2 trial results.

## Review

### How should we apply the results from the CRASH-2 trial?

Some clinicians believe that a treatment should only be used in patients similar to those included in the trial (or trials) that showed the treatment to be effective. In their paper ‘Tranexamic acid in trauma: How should we use it?’ Neapolitano et al. recommend that TXA is used only in adult trauma patients, presumably because only adults were included in the CRASH-2 trial [6].Their advice is not without potential consequences. Every year, worldwide, tens of thousands of children bleed to death after trauma and if TXA is also safe and effective in children, it could prevent hundreds of child deaths. Similarly, in arguing for a new trial of TXA in Australasian patients, Gruen et al. point out that many of the patients in CRASH-2 trial were from middle income countries and ‘because substantial differences are likely between advanced and less developed trauma systems, hypotheses about TXA should be reinvestigated.’ Gruen calls for more evidence on the benefits and risks of TXA in patients treated to ‘modern civilian and military trauma standards’ [4].

We do not deny the existence of international differences in trauma care, but this does not necessarily mean that results obtained in one setting cannot be applied in another. Nor does the failure to include children in the CRASH-2 trial mean that the results cannot be generalised to children. The view that generalisation is a simple extrapolation from a trial population to a patient population confuses statistical and scientific inference [7]. Whereas statistical inference, the process of using sample information to make inferences about the population from which it was drawn, is helped by having a representative sample; scientific inference involves making valid conclusions about how biology works, and important insights into the effects of drugs can be obtained from patients that are completely unrepresentative of the patients in which the drug might be used. Children were excluded from the CRASH-2 trial, not because the investigators did not intend the results to be applied in children, but because it is logistically difficult to conduct large trials that include children, especially in emergency situations. For example, using a fixed dose of a treatment is more practical than a body weight-related dose. With a fixed dose, treatment instructions can be simplified and there is no need to weight the patient and make a calculation, which in an emergency is time consuming and error prone. However, fixed doses cannot be used in children, and so the most efficient way to obtain valid and precise estimates of the effect of TXA in people of all ages was to conduct a large trial that excluded children.

To generalise results, we must consider the likely mechanism by which the treatment affected the health outcome and the factors that might be relevant to this mechanism [7]. The observation, made in 1954, that smoking increases mortality in male British doctors, was readily extrapolated to smokers in general, even though the doctors studied were unrepresentative of a wider population in terms of sex, age, social class and ethnicity [8]. Being male, being British and being a doctor are not relevant to the biological effect of smoking. Similarly, when deciding which trauma patients should be treated with TXA, we must consider how TXA reduces the risk of death due to bleeding and what patient characteristics might be relevant to the mechanism of action. Is there any biological reason why TXA might work differently in children or in patients receiving ‘modern’ trauma care?

### Mechanism of action of tranexamic acid

The best evidence that TXA reduces bleeding is from randomised controlled trials of TXA in surgery where, unlike trauma, bleeding can be measured reasonably accurately. A 2012 systematic review and meta-analysis, which included data from 104 trials with a total of 8,030 patients, showed that TXA reduces bleeding by about one third (pooled ratio = 0 · 66, 95% CI 0 · 65–0 · 67; *P* < 0 · 001) regardless of the type of surgery and the extent of bleeding [9,10]. There were also fewer deaths in TXA-treated patients (0.61, 0.38–0.98; *P* = 0.04), although when the analysis was restricted to trials using adequate allocation concealment there was more uncertainty (0.67, 0.33–1.34; *P* = 0.25).

Tranexamic acid reduces bleeding by inhibiting the enzymatic breakdown of fibrin blood clots. Plasminogen, a glycoprotein pro-enzyme produced by the liver, is converted into the fibrinolytic enzyme plasmin by tissue plasminogen activator (TPA). The plasminogen molecule is folded into loops called kringles that protrude like fingers. Plasminogen binds to fibrin via lysine-binding sites situated on the tips of the kringles [11,12]. If the lysine residues on fibrin are enzymatically removed, the binding of plasminogen is inhibited. Fibrin binds both plasminogen and TPA thus localizing and enhancing plasmin formation [11]. Plasmin that is bound to fibrin is also less susceptible to plasmin inhibitors. Plasmin splits fibrin into fibrin degradation products. This exposes more lysine residues which bind more plasminogen, thus accelerating fibrinolysis. TXA is a molecular analogue of lysine that inhibits fibrinolysis by reducing the binding of plasminogen to fibrin.

Although new discoveries in molecular biology and clinical research may elaborate the mechanism outlined above, based on current knowledge, the causal chain linking TXA to reduced mortality includes inhibition of plasminogen binding, less plasmin formation, decreased fibrinolysis, less bleeding and fewer exsanguinations. The question for doctors providing ‘modern’ trauma care is what aspects of contemporary care are relevant to this (or some other hypothesised) mechanism of action? Although Gruen et al. mention ‘rapid access to blood products, damage control surgery and angiography, and advanced critical care’ as factors, they give no biological reason why these treatments would affect the mechanism of action of TXA such as to mitigate its effect. Similarly, is there any reason why TXA would stop an 18-year old from bleeding to death but not an 8-year old? A working group set up by the UK Royal College of Paediatrics and Child Health, aware of the evidence that TXA reduces bleeding in paediatric surgery, was prepared to generalise the CRASH-2 trial results to paediatric trauma [13]. They recommended that an adult TXA dose is used in children over 12 and a weight-related dose for younger children.

### How much benefit should we expect from TXA treatment?

#### Do we generalise the risk difference or the risk ratio?

The CRASH-2 trial compared the risk of death in TXA and placebo allocated patients. There are two commonly used measures of the effect of treatments: the risk difference and the risk ratio. Neapolitano et al. focus on the risk difference and are unimpressed: ‘What is critical is the modest effect on the overall population: All-cause mortality was “significantly” reduced from 16.0%–14.5% (NNT, 67). The risk of death caused by bleeding overall was “significantly” reduced from 5.7%–4.9% (NNT, 121)’ [6]. Their concern is that a risk difference of only 0.8% (5.7%–4.9%), whilst statistically significant might be clinically unimportant. But is the risk difference a generalizable measure?

**Figure 1:**
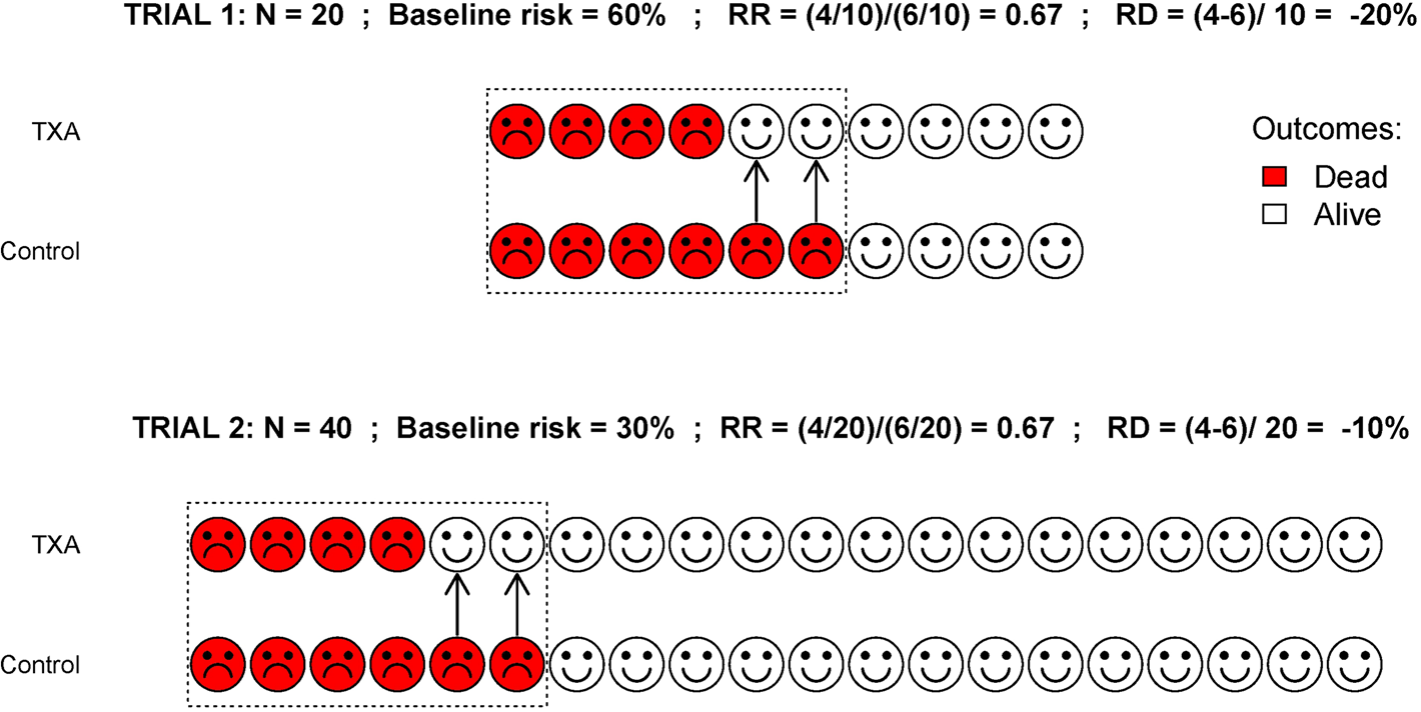
**Two hypothetical trials: the risk ratio is the generalizable measure of the effect of treatment.** In both trials, the biological effect of the treatment is the same, corresponding to a one third reduction in the risk of death. The relative risk is the same in both trials and reflects the biological effect of the treatment. However, the risk difference is not the same. The relative risk is a generalizable measure of the biological effect of a treatment, whereas the risk difference is not generalizable.

Figure 1 shows two hypothetical clinical trials. The first compared the risk of death due to bleeding (red circles) in ten TXA-treated and ten control patients. There were four deaths in the TXA group and six deaths in the control group. The purpose of the control group in a randomised controlled trial is to indicate what would have happened in the absence of treatment. In this case, two of the six patients (one third) who would have died if untreated, were saved by TXA. In biological terms, TXA blocked the pathophysiological mechanism leading to death in one third of the patients who would otherwise have died. The risk of death in the control and TXA groups was 60% and 40%, respectively. The risk difference is 20% and the risk ratio is 0.67. The second trial compared the risk of death due to bleeding in 20 TXA-treated and 20 control patients. Again, there were four deaths in the TXA group and six deaths in the control group. Once again, two of the six patients who would have died if untreated, were saved by TXA. The biological effect is the same in both trials. The risk of death in the control and TXA groups was 30% and 20%, respectively. This time the risk difference is 10%, although the risk ratio is again 0.67. The biological effect of a treatment is indicated by the risk ratio. Indeed, the relative risk reduction (1-risk ratio) is the proportion of patients in whom the mechanism leading to death is blocked by treatment.

When we generalise results, we do so on the basis that the biological effect of treatment will be broadly similar in the patients in whom the treatment will be used [7].The risk ratio is therefore the generalizable measure. If the number of patients is the same in the treated and control groups, the risk ratio is simply the ratio of outcome events in the two groups. Unsurprisingly, only patients who experience the outcome can contribute information on the effect of the treatment on that outcome. The risk difference on the other hand, depends on the number of patients who did not experience the outcome. Furthermore, because there is no reason to expect that any given patient would have the same baseline risk as the patients in the trial, the risk difference is not generalizable. The number needed to be treated, which is the reciprocal of the risk difference, is also not generalizable. Instead it should be calculated by applying the risk ratio from the trial to the patient’s baseline risk. If the baseline risk of death is estimated at 30% and the risk ratio is two thirds (i.e. RR = 4/6), we would expect 20% of treated patients to die. The risk difference is 10% and the number needed to treat is 10. When given soon after injury, TXA reduces the risk of bleeding to death by about a third. It would be hard to argue that a one-third reduction in the risk of bleeding to death is unimportant.

#### All-cause mortality or cause-specific mortality?

When given within one hour of injury, there was a substantial reduction in death due to bleeding (RR = 0.68) that was highly statistically significant but no reduction in non-bleeding deaths (RR = 1.04). There was also a statistically significant reduction in all-cause mortality (RR = 0.87). On the basis of these results, by how much would we expect TXA to reduce mortality in other trauma patients? Can we expect a similar reduction in death from bleeding, all-cause mortality or in both of these measures?

**Figure 2:**
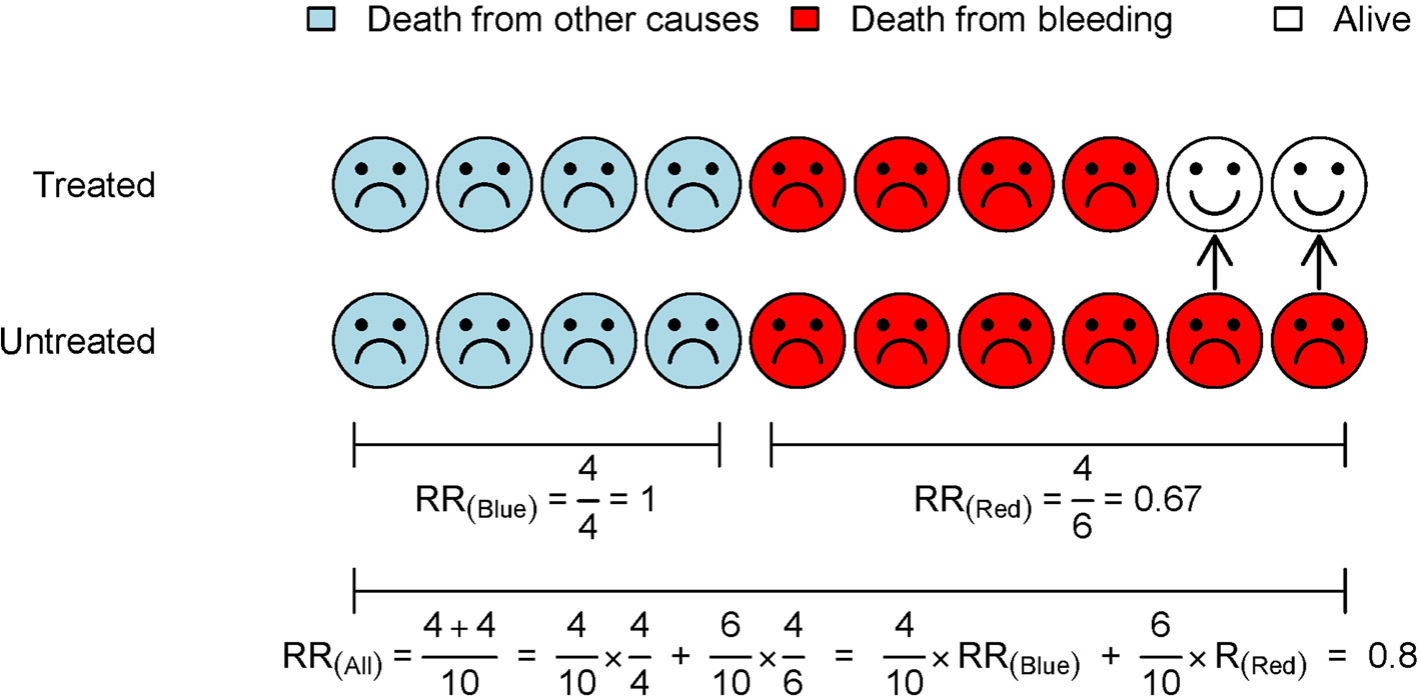
**Hypothetical trial: effect on all-cause mortality is a weighted average of effect on cause-specific mortality.** The treatment reduces the risk of death due to bleeding by one third (RR = 0.67) but has no effect on other causes of death (RR = 1.0). The effect on all-cause mortality (RR = 0.8) is a weighted average of its effect on deaths due to bleeding and on other causes of death, weighted according to the relative contributions of the different causes.

We have seen that the risk ratio for death is essentially the ratio of deaths in the treated and control groups. Figure 2 shows results from a hypothetical clinical trial of treatment for bleeding. There is a one-third reduction in deaths due to bleeding but no effect on other causes of death. The effect on all-cause mortality is an average of its effect on specific causes of death, weighted according to the relative contributions of the specific causes. In the CRASH-2 trial, 44% of deaths within an hour of injury were due to bleeding and 55% were from other causes in the control group. The overall effect on all-cause mortality is a weighted average of the effect on bleeding and non-bleeding deaths: 0.68 × 0.44 + 1.04 × 0.55 = 0.87. However, the relative contribution of causes of death in the population in which the results are being applied might be different to that in the trial. For example, in the CRASH-2 trial data, about 60% of patients with penetrating trauma died from bleeding compared with 25% of patients with blunt trauma. Consequently, the effect of TXA on all-cause mortality is not generalizable. On the other hand, the effect of TXA on death due to bleeding should be generalizable since this reflects the biological mechanism of action of TXA which we would expect to be broadly similar (i.e. generalizable) in different patients.

### Is tranexamic acid more or less effective in particular groups of patients?

An awareness of the circumstances necessary for the valid generalisation of treatment effects is the essence of therapeutic knowledge [14]. Subgroup analyses of clinical trials can sometimes deepen our understanding of these circumstances and suggest whether the treatment is more or less effective in particular patient groups. However, subgroup analyses are often misleading. Chance alone can produce spurious subgroup results that can lead to patients being denied an effective treatment [15]. On biological grounds, we should expect treatments to have broadly similar effects on a given outcome and should be sceptical of claims that a treatment is beneficial in one subgroup but harmful in another. Indeed, differential subgroup effects are more credible if based on a sound biological rationale that is precisely specified prior to data analysis [15]. Patient characteristics can vary widely but only those that are relevant to the mechanism by which the drug prevents the particular adverse outcome should lead to differential effects. What biological reason is there to expect a larger or smaller proportion of outcome events to be affected by the treatment? The risk of inappropriate inferences can be reduced by using the overall relative risk reduction in the trial as the best estimate of the likely effect in each subgroup, only departing from this assumption if there is strong evidence to the contrary. In other words, the average biological effect of the treatment that was observed in the trial (or meta-analyses of all relevant trials) is the best guide to its effect in any particular subgroup [15].

Although there was no evidence that the effect of TXA varied according to baseline systolic blood pressure (*P* = 0.33), after pointing out that ‘the signal for benefit of TXA was in the most severe shock group as defined by admission SBP’ Neapolitano and colleagues advised that TXA is used only in patients ‘with severe haemorrhagic shock (SBP < 75 mm Hg)’ [6]. The focus on this subgroup is hard to understand from a biological perspective and could have serious implications for patient care. Since TXA reduces bleeding and early treatment is most effective, it seems sensible to give TXA before the blood pressure falls to dangerously low levels. TXA appears to reduce the risk of death due to bleeding by about one third, regardless of the patient’s baseline risk [16]. If the patient’s risk of bleeding to death is 30%, giving TXA should reduce the risk to about 20%; and if the baseline risk is 3%, then TXA should reduce this to about 2%. Because there are many more patients at low risk, a policy of giving TXA to all bleeding trauma patients would prevent more deaths than using it only in the most severely injured. Indeed, using data from a large UK-based trauma registry, we showed that if TXA use is limited to patients with a baseline risk of more than 20%, we would miss most of the benefit, since 53% of TXA preventable deaths are in patients who had a baseline risk of death of less than 20% [16].

In the CRASH-2 trial, we prespecified an analysis of the effect of TXA by time from injury to the start of treatment (<1, 1–3, 3–8 h). We hypothesised that TXA would be most effective soon after injury, when bleeding is profuse, and less effective later, when the acute phase response to trauma increases the risk of thrombosis [17]. We found strong evidence in support of our hypothesis (*P* < 0.0001). In patients treated within 3 h, TXA substantially reduced the risk of death due to bleeding but after 3 h, TXA appeared to increase the risk of death due to bleeding [3]. Time from injury is almost certainly a proxy for a change in the pathophysiological state of the patient relevant to the mechanism by which TXA reduces bleeding. We think this could be PAI-1-induced suppression of fibrinolysis and the onset of thrombotic disseminated intravascular coagulation (DIC) [18,19]. DIC is characterised by intravascular activation of coagulation with widespread fibrin deposition. Because TXA inhibits fibrinolysis, it could exacerbate this process. Although the underlying pathology is thrombosis, due to the consumption of coagulation factors, thrombotic DIC usually manifests as bleeding. This might explain why TXA appeared harmful when initiated after 3 h. Late treatment with TXA may have increased the risk of thrombotic DIC. Among patients treated after 3 h, deaths apparently due to bleeding may have been caused by intravascular thrombosis, a completely different mechanism of death. Further research may help to explain this striking subgroup result.

If we could identify only those patients who will benefit from TXA treatment, we could avoid treating patients unnecessarily. This understandable objective may explain why some authors recommend that TXA is used only in patients with evidence of ‘hyperfibrinolysis’ on thromboelastography [6,20]. After all, if TXA works by inhibiting fibrinolysis, it should not be effective when fibrinolysis is absent. However, fibrinolysis is not present or absent, but present to varying degrees, and even patients with ‘normal’ levels of fibrinolysis may benefit from TXA if this reduces bleeding [21]. The degree of fibrinolysis is not the only factor that determines the risk of death. The extent of the bleeding and other patient factors are also important. Age is a strong risk factor for death in trauma patients [22]. Older patients appear less able to tolerate blood loss. Even a slight reduction in bleeding could prevent death in patients who are at high risk for other reasons. Trials of TXA in surgery show that TXA reduces bleeding by about one third even when bleeding is modest [10]. The challenge for clinicians is not to identify patients with ‘hyperfibrinolysis’, but to identify patients at risk of bleeding to death who might benefit from bleeding less. By way of analogy, although cholesterol reduction is the mechanism by which statins reduce the risk of myocardial infarction, this does not mean that statins should only be used in patients with high cholesterol levels. Statins also reduce the risk of myocardial infarction in patients with ‘normal’ cholesterol levels [23]. Treatment decisions should be based on the risk of the adverse outcome (prognosis) rather than an arbitrary diagnosis based on a single parameter. Since TXA is highly cost-effective, with no serious side effects, it could be used in all trauma patients at risk of bleeding to death [24].

## Conclusions

When generalising results from clinical trials, biological insight into the mechanism of action of the treatment and an awareness of the circumstances in which a finding applies are more relevant to patient care than the application of statistical reasoning. A deeper consideration of biological mechanisms should also result in wider recognition that the risk ratio is the generalizable measure of the treatment effect and that differential subgroup effects should be viewed with scepticism.

The ultimate aim of therapeutic research is to formulate general conclusions about the effects of medicines that can inform the management of different patients, in different places and different times. This process of scientific inference is greatly aided by results from studies that rigorously control bias and random error, which usually implies randomisation of large numbers of patients (thus providing large numbers of outcome events). It is not necessary, however, that the trial population is representative of the population in which the medicine will be used.

On the other hand, statistical inference, the process of using sample information to reach conclusions about the population from which it was drawn, is helped by having a representative sample. If we wanted to know what proportion of trauma patients received TXA, we might conduct a survey in a representative sample of trauma patients. The results would apply to a specific location at a particular time. The reluctance to generalise treatment effects on the basis of representativeness confuses scientific and statistical inference [6]. It is important to emphasise that we do believe that further trials of TXA in trauma patients are needed. However, future trials should aim to extend our knowledge of the effects of TXA and should therefore address important biologically based treatment uncertainties.
